# Association between Self-Medication for Mild Symptoms and Quality of Life among Older Adults in Rural Japan: A Cross-Sectional Study

**DOI:** 10.3390/medicina58060701

**Published:** 2022-05-25

**Authors:** Ryuichi Ohta, Yoshinori Ryu, Chiaki Sano

**Affiliations:** 1 Community Care, Unnan City Hospital, 699-1221 96-1 Iida, Daito-cho, Unnan 699-1221, Japan; yoshiyoshiryuryu.hpydys@gmail.com; 2 Department of Community Medicine Management, Faculty of Medicine, Shimane University, 89-1 Enya cho, Izumo 693-8501, Japan; sanochi@med.shimane-u.ac.jp

**Keywords:** community care, comprehensive care, help-seeking, Japan, older adults, quality of life, rural communities, self-medication

## Abstract

Self-medication, a help-seeking behavior to control individual symptoms, can be promoted to prevent the overuse of medical care and improve self-management among older adults. However, evidence regarding the association between self-medication and quality of life (QOL) is lacking. The purpose of this study is to investigate the association between QOL and the usage of self-medication among rural older adults. This cross-sectional study included participants older than 65 years in rural Japanese communities. Data were collected using a questionnaire regarding self-medication trends, the EQ-5D-5L to assess QOL, and a demographics questionnaire. Participants were divided into exposure and control groups based on their tendencies toward self-medication usage. Differences in the demographics between groups were adjusted using propensity score matching. Results: The health status in the exposure group was statistically significantly better than that in the control group in the dimensions of movement, self-care, and usual activities. Conversely, the pain/discomfort and anxiety/depression dimensions were not statistically significantly different. The quality of self-medication behaviors for mild symptoms can be improved with practical knowledge of and access to home remedies and over-the-counter drugs. Educational interventions and system development for better self-medication for mild symptoms and medical care for critical symptoms in rural contexts can be effective in improving QOL among rural older adults.

## 1. Introduction

Aging societies must contend with increasing medical issues among older adults, which, in turn, needs dedicated health management. Research has indicated that older adults over 65 years of age develop numerous medical diseases that require management and the use of established healthcare systems [1,2]. The increase in the number of medical treatments and visits to medical institutions leads to increased medical costs and the burden of medical care, which can cause financial problems for older adults [3]. To avoid the overuse of healthcare resources, older adults may need effective health management and lay care [4,5]. In this context, effective help-seeking behaviors (HSBs) are critical [6,7,8]. HSBs are divided into lay and professional care, and both should be used effectively to control health conditions [9]. Lay care refers to care provided by lay people, or those who have received no formal training and are not paid, such as self-care and care from relatives, friends, and self-help groups. Conversely, professional care refers to care provided by trained and paid professionals, usually in formal settings [9].

Self-medication is a type of lay care HSB that controls older adults’ symptoms and can be promoted to reduce medical care usage. It refers to the use of home remedies, over-the-counter drugs, and previously prescribed medicines to treat symptoms [10]. It is essential for managing health conditions and can efficiently reduce the utilization of professional care for treating mild symptoms [10,11]. Over 70% of older adults have chronic diseases causing mild symptoms that can be mitigated through self-medication [12,13,14]. Mild symptoms refer to symptoms that people feel are manageable without healthcare support. However, severe symptoms need healthcare support immediately [9]. Therefore, self-medication is common among older adults with mild symptoms and can have both advantages and disadvantages for their health. Self-medication for severe symptoms can potentially delay medical care and deteriorate the symptoms, causing critical situations, longer admission times, and more morbidity and mortality for people [12,13,14]. Appropriate knowledge of over-the-counter drugs and home remedies is essential for effective self-medication for mild symptoms. The use of over-the-counter drugs, which could be bought in drug stores, and home remedies, which could be made in homes with natural resources, may be related to age, sex, educational level, social support, social capital, and place of residence [12,13,14]. Effective self-medication for mild symptoms can mitigate the symptoms and should be improved to ensure the prevention and appropriate usage of healthcare for diseases among older adults.

Specifically, self-medication for mild symptoms needs improvement in rural areas where people are far from various resources and cannot approach healthcare resources, professionals, or people who have appropriate knowledge of HSBs. Rural settings affect the health conditions of rural older adults [15,16]. As health insurance compensates most medical costs in Japan, many older adults in urban areas frequently use medical institutions, even if their symptoms are mild [17,18]. However, due to a lack of health resources and a low number of relatives nearby, rural older adults may need to self-manage their conditions and visit medical institutions without consulting others [19,20]. In relation to this reality, two studies have attempted to comprehensively provide information regarding self-medication via various information resources to improve the knowledge of and skills for self-medication among rural older adults and help them to safely perform self-medication [21,22].

The rural health care resources and potential usage conditions point to the possibility that efficient self-medication is necessary for effective health management and control of mild symptoms among the rural older adult population. However, researchers have also indicated that overuse of self-medication can negatively affect health [21,22]. Furthermore, although some studies have indicated that improvements in the quality of self-medication behavior may be associated with better health [22,23], evidence regarding the association between self-medication and quality of life (QOL) is lacking. Health conditions could be measured as objective or subjective. Objective health could be measured by clinical data, such as blood pressure and other laboratory tests. Subjective health cold be measured based on patients’ perception of their health. QOL is defined as a person’s perception of their conditions in their lives in the context of their culture and value systems. QOL is an important concept of subjective health and is measured through questionnaires regarding usual conditions in life [7]. Individuals in rural areas may also lack access to healthcare because of limited resources. Therefore, effective self-medication is critical for them [16]. There is a lack of appropriate interventions in Japan for enhancing the knowledge of self-medication for mild symptoms among older adults living in rural areas and getting help for critical symptoms from healthcare professionals, in addition to a lack of interest from stakeholders [15,16]. Evidence concerning the association between self-medication and rural older adults’ subjective health may illustrate the importance of improving self-medication behaviors in this population.

Hence, this study’s research question is: “Is self-medication for mild symptoms associated with high QOL in rural Japan?” By elucidating this association, the relationship between effective self-medication and better health conditions among the rural older adult population can be considered. Furthermore, the adverse effects of self-medication on QOL have not been studied. We hypothesize that an increase in appropriate self-medication can mitigate the burden on medical professionals. Conversely, higher QOL and other socioeconomic factors can increase older adults’ use of self-medication. Therefore, the purpose of this study is to investigate the association between QOL and the usage of self-medication among rural older adults.

## 2. Materials and Methods

This cross-sectional study was performed to investigate the association between QOL and the usage of self-medication among participants older than 65 years in rural Japanese communities.

### 2.1. Setting

Unnan City is a rural city in southeast Shimane Prefecture, Japan. A 2017 survey revealed that its total population was 38,882 (18,720 males and 20,162 females), with 37.82% being older than 65 years. This proportion is estimated to reach 50% by 2050. Unnan City has 30 autonomous communities that perform activities to improve people’s QOL [16]. Each autonomous community is allowed to conduct various local activities designed to solve specific social problems. Each autonomous community employs community workers who are skilled at communicating with older citizens to communicate with citizens in addition to coordinating and managing activities. Their activities are financially supported and monitored by their respective cities [15]. This study was performed in Kakeya and Yoshida towns, which are in the southwestern part of Unnan City and encompass a total of six autonomous communities (Kakeya, Matsukasa, Tane, Iruma, Hata/Namami, Yoshida, and Tai). Each town has one primary care clinic containing one physician. Participants from four autonomous communities (Kakeya, Tai, Matsukasa, and Tane) were included in this study.

### 2.2. Participants

Of the study population, 572, 247, 129, and 211 older adults resided in Kakeya, Tai, Matsukasa, and Tane, respectively. Participants were informed about this study through a letter, which comprised of an explanation and a research questionnaire in Japanese. The inclusion criteria were individuals over 65 years old who lived in the four autonomous communities: Kakeya, Tai, Matsukasa, and Tane. Individuals with inadequate reading or writing skills in Japanese and those with cognitive dysfunction or dementia based on their past medical histories informed by either participants or their families were excluded from this study.

For the sample size calculation based on a previous study, the minimally important change in the health status index scores of the EQ-5D-5L was estimated to be 0.04 [24]. To clarify the statistical difference in the EQ-5D-5L health status index scores of 0.04 (standard deviation = 0.11), a minimum of 126 participants were required in both the exposure and control groups, with an alpha error of 0.05, a beta of 0.20, and a power of 80% based on GPower.

### 2.3. Measurements

A questionnaire was administered to all participants from December 2020 to January 2021. The researchers collaborated with the local autonomous community organization, which had community workers stationed in each region. Each region’s community workers distributed the questionnaires to participants and collected them upon completion. Each community worker was instructed by the researchers and performed the process of collecting the questionnaire to ensure good scientific conduct.

### 2.4. Self-Medication

A validated questionnaire was used to assess participants’ care preferences for mild symptoms [9]. This questionnaire was established to investigate older adults’ HSBs for mild symptoms. This questionnaire contained 12 choices for the responses to their potential HSBs for mild symptoms. In the questionnaire, participants could choose multiple potential behaviors when they had mild symptoms. The questionnaire’s validity and reliability were checked between their choices in the questionnaire and their real-life help-seeking behaviors [9]. In this questionnaire, the participants choose whether they use home remedies or over-the-counter drugs when they have mild symptoms. The questionnaire did not check whether the self-medication was based on advice from medical professionals. In Japan, over-the-counter drugs are regularly sold to residents by local pharmaceutical companies. Using the results of the questionnaire, participants were divided into the exposure (self-medication) and control groups. The exposure group was defined as participants who used home remedies or bought over-the-counter drugs when they had mild symptoms; the control group was defined as those who did not use home remedies or buy over-the-counter drugs when they had mild symptoms.

### 2.5. Primary Outcome 

QOL was measured as a health status index score using the EQ-5D-5L questionnaire, which is a self-reported questionnaire comprising of five dimensions of health states (mobility, self-care, usual activities, pain/discomfort, and anxiety/depression) [24,25]. Responses for each dimension were rated using five levels of severity: no problems (1 point), slight problems (2 points), moderate problems (3 points), severe problems (4 points), and extreme problems (5 points). The responses to the five dimensions were combined into a five-digit number indicating each participant’s health status profile, which was converted into a single health status index score by applying a formula that attached values to each response. A Japanese version of the questionnaire, which has been validated, was used in this study [25].

### 2.6. Other Variables

The questionnaire also measured factors related to self-medication, including age, sex, body mass index (BMI), smoking habits, alcohol intake, education (graduation from high school or not), living with family, social support [26], social capital (10-point Likert scale) [27], and socioeconomic status (SES) (four-point Likert scale). The following variables were categorized binomially: social capital (high [10 to 6] = 1, low [5 to 1] = 0) and SES (high [rich or relatively rich] = 1, low [poor or relatively poor] = 0).

### 2.7. Statistical Analysis

The data regarding age and BMI were analyzed by performing a student *t*-test. Categorical data regarding smoking habits, alcohol intake, education, living with family, social support, social capital, and SES were analyzed by performing a chi-squared test. Propensity score matching was performed when adjusting for differences in the demographics between the exposure and comparison groups. Propensity scores were calculated using the demographic variables of age, sex, BMI, smoking habit, alcohol intake, education, living with family, social support [26], social capital, and SES. One-to-one caliper matching was used with no replacement to match the exposure and control groups. Next, the covariate balance between the matched groups was examined. After the adjustment, participants’ primary outcomes were compared between the two groups by performing a student t-test. A significance level of *p* < 0.05 was set for all comparisons. An easy *R* and a graphical user interface for *R* were used for the statistical analyses [28].

### 2.8. Ethical Considerations

The participants were informed about the research aims, how their data would be disclosed, and how their personal information would be protected. Subsequently, they provided written informed consent. This study was approved by the Unnan City Hospital Clinical Ethics Committee (approval code 20190036). The investigations were performed following the rules of the 1975 Declaration of Helsinki, revised in 2013.

## 3. Results

### 3.1. Participants’ Demographic Data 

**Figure 1:**
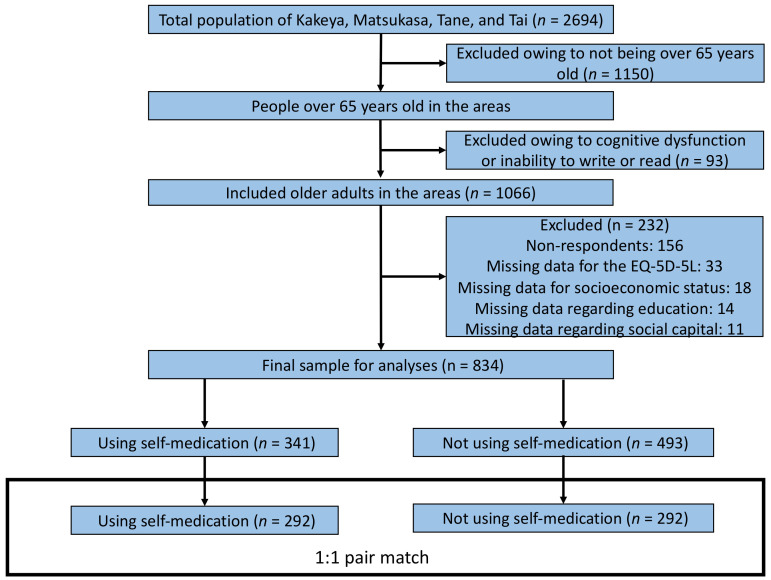
Participant selection process.

Figure 1 portrays a flowchart of the sample selection process. The total population of Kakeya, Matsukasa, Tane, and Tai was 2694 from the Japanese national data. Among these individuals, 1159 were older than 65 years, and 93 were excluded because of their inability to read or write appropriately or having cognitive dysfunction or dementia. A total of 1066 participants agreed to participate in this study and were provided with the questionnaire; 232 were excluded due to a lack of response and missing data regarding the health status index scores, SES, education, and social capital. Finally, through propensity score matching, 584 participants were selected for final analysis.

Participants in the exposure group were younger (*p* < 0.001), had fewer chronic diseases (*p* < 0.001), and had higher levels of education (*p* = 0.001) than those in the control group. Additionally, there were more participants in the exposure group who smoked and had greater social support than those in the control group (*p* = 0.012 and 0.032, respectively). No statistically significant differences were found in terms of sex, BMI, alcohol intake, living with family, annual health checkups, and social capital (Table 1).

After adjusting for propensity score matching, 292 participants in the exposure group were matched with 292 participants in the control group. The C-statistic for the propensity score regression model was 0.625 (95% confidence interval [CI], 0.585–0.664). After adjustment, no statistically significant differences were observed between the exposure and control groups (Table 1).

### 3.2. Association between the Preference for Self-Management, Health State, and Health Status Index Scores

After propensity score matching, the health status index scores for the EQ-5D-5L were statistically higher in the exposure group than those in the control group. The health state levels in the exposure group were statistically significantly higher in the dimensions of movement, self-care, and usual activities than those in the control group. However, the health state levels in the pain/discomfort and anxiety/depression dimensions were not statistically significantly different between the groups (Table 2).

## 4. Discussion

This study indicates that the trend of using self-medication for mild symptoms is associated with high QOL among rural older adults. In particular, this population had higher scores in the dimensions of mobility, self-care, and usual activities, which supports the study hypothesis. This positive association may indicate that improvement in the quality of self-medication could contribute to improving older adults’ subjective health. The relationship between these factors should be investigated in future studies. In rural contexts that lack healthcare resources, rural older adults’ self-medication behavior can be effectively promoted by improving their understanding of home remedies and over-the-counter drugs, potentially contributing to enhancing their QOL.

Research has indicated that the use of self-medication to treat mild symptoms and high QOL are largely connected and may affect each other. For example, studies have indicated that rural older adult populations with preferences for self-medication have higher scores for certain QOL dimensions, such as satisfaction and decreased anxiety [29,30]. Conversely, older adults with high QOL have been found to address mild symptoms by using self-medication [31]. Further, because these older adults with high QOL may have a stronger foundation in health literacy, they may seek professional care if their symptoms persist [32].

Considering that older adults with high QOL may have adequate or high health literacy, they may be able to assess their symptoms through effective self-management, which may facilitate the use of self-medication. Therefore, high health literacy may be associated with subjective and objective health [33,34,35]. Further, people with high QOL and health literacy may perform effective self-medication based on their health literacy [22], which is essential for symptom management [36,37]. In Japan, various HSB information regarding self-medication and self-management could be accessed from the internet and leaflets from the government. People with high QOL can manage their symptoms through various HSBs based on their knowledge, including self-medication support and effective self-management of mild symptoms [36,37].

More importantly, rural older adults’ preference for self-medication can affect their confidence and self-efficacy in health management. Self-efficacy refers to people’s belief in their abilities to perform behaviors necessary to accomplish specific aims. When older adults are confident about their self-health management abilities through education on self-management and self-medication, they may use these skills to maintain their health and manage their symptoms [38,39,40]. This can improve their self-efficacy regarding symptom management [41] and increase their preference to manage mild symptoms through self-medication [42,43]. Additionally, effective health management with appropriate health knowledge can improve the overall perception of health, and lead to higher subjective health [40,44]. Since this study used a cross-sectional design, longitudinal and interventional studies are warranted to explore older adults’ knowledge and skills regarding self-medication and investigate the relationship between self-management and subjective health.

Regarding recommendations for self-medication interventions, both our findings and the prior research indicate the need to respect the medical care context in which the intervention will be applied. Effective use of self-medication can mitigate the overuse of primary care and emergency medicine, particularly among older adults [3,45]. Therefore, education regarding HSBs for mild symptoms is needed [3,45]. Moreover, for the assessment of the effective usage of self-medication, health care professionals and community workers should monitor people’s concrete self-medication. Further, HSBs can be associated with patients’ backgrounds and contexts, including personal factors (e.g., medical resources and knowledge of health management) and community factors (e.g., community social norms, which inhibit effective self-medication) [42,46,47]. These factors should be considered when developing interventions for HSBs. Accordingly, effective self-medication interventions for older adults in rural areas should be based on a careful assessment of their healthcare needs and should respect the balance between their needs and the healthcare capacity in their areas.

This study has certain limitations. Firstly, its cross-sectional design did not allow for the examination of the cause-and-effect relationship between self-medication and QOL. Secondly, this study did not assess the adverse effect of self-medication on QOL. Longitudinal studies are recommended to clarify the relationship between self-medication and QOL, including the adverse effects of self-medication. Thirdly, as we conducted our study in super-aged rural communities in Japan, our sample had selection bias. To overcome this limitation, we collected data from a broad range of communities. Japan is experiencing the aging population phenomenon, and many other developing and developed economies will soon follow this trend. Thus, our findings may prove useful for such communities. Future studies could be conducted in other urban and rural areas with different cultures and social norms to demonstrate the validity of our results. Finally, the definition of self-medication varies. We used the variables from the questionnaire that have been validated for use in the Japanese rural context to ensure greater reliability. This questionnaire also focused on mild symptoms perceived by participants. To assess the possibility of self-medication for critical symptoms and the effectiveness of over-the-counter drugs, future research should investigate the association between QOL and self-medication in terms of critical symptoms and the participants’ perceptions of the usage of over-the-counter drugs and home remedies. Future studies could also clarify the best methods of self-medication among older adults in rural areas with limited medical resources.

## 5. Conclusions

The use of self-medication in HSB for mild symptoms can be associated with high QOL among older adults. Practical knowledge of and access to home remedies and over-the-counter medicine can be critical for improving the quality of self-medication behaviors. Educational interventions and system development to enable better self-medication for mild symptoms and healthcare usage for critical symptoms can be effective in improving QOL among older adults in rural contexts.

## Figures and Tables

**Table 1:** Demographic characteristics for each group and significance level of the comparison between the two groups before and after propensity score matching.

	Crude			After Propensity Score Matching		
	Exposure	Control	*p*-Value	Exposure	Control	*p*-Value
Variables	*n* = 341	*n* = 493		*n* = 292	*n* = 292	
Age (in years), mean (SD ^1^)	75.86 (±8.15)	78.80 (±7.69)	<0.001	76.50 (±7.88)	76.50 (±7.46)	1
Sex, male (%)	159 (46.8)	207 (42.0)	0.178	130 (44.5)	124 (42.5)	0.676
BMI, mean (SD)	22.52 (±3.35)	22.71 (±4.07)	0.483	22.64 (±3.33)	22.45 (±3.61)	0.523
Having chronic diseases (%)	270 (79.2)	450 (91.3)	<0.001	251 (86.0)	253 (86.6)	0.904
Alcohol (%)	129 (38.2)	155 (31.6)	0.053	104 (35.6)	108 (37.0)	0.796
Tobacco (%)	34 (10.0)	27 (5.5)	0.012	19 (6.5)	22 (7.5)	0.746
Higher education (%)	175 (51.6)	197 (40.3)	0.001	147 (50.3)	144 (49.3)	0.869
Living with family (%)	299 (89.3)	422 (86.5)	0.282	257 (88.0)	257 (88.0)	1
Annual health check-up (%)	242 (71.2)	354 (72.4)	0.753	214 (73.3)	206 (70.5)	0.519
High SES (%)	161 (47.6)	223 (45.9)	0.67	135 (46.2)	134 (45.9)	1
High social capital (%)	284 (85.0)	408 (84.1)	0.769	248 (86.4)	249 (85.9)	0.904
High social support (%)	270 (80.8)	427 (86.6)	0.032	240 (82.2)	243 (83.2)	0.827

^1^ SD: standard deviation.

**Table 2:** Comparison of health status index scores and health state levels for the five dimensions of the EQ-5D-5L before and after propensity score matching.

	Crude			After Propensity Score Matching		
	Using Self-Medication	No Use	*p*-Value	Using Self-Medication	No Use	*p*-Value
EQ-5D-5L	*n* = 341	*n* = 493		*n* = 292	*n* = 292	
Health status index scores, mean (SD ^1^)	0.72 (±0.16)	0.67 (±0.22)	<0.001	0.72 (±0.16)	0.68 (±0.22)	0.024
Health state levels						
Mobility, mean (SD)	1.57 (±0.91)	2.00 (±1.31)	<0.001	1.60 (±0.93)	1.91 (±1.28)	0.001
Self-care, mean (SD)	1.29 (±0.69)	1.61 (±1.10)	<0.001	1.28 (±0.69)	1.52 (±1.00)	0.001
Usual activities, mean (SD)	1.65 (±0.87)	1.96 (±1.11)	<0.001	1.66 (±0.88)	1.88 (±1.08)	0.007
Pain/discomfort, mean (SD)	2.15 (±0.84)	2.35 (±1.02)	0.002	2.19 (±0.86)	2.30 (±1.03)	0.138
Anxiety/depression, mean (SD)	1.73 (±0.79)	1.86 (±0.95)	<0.049	1.75 (±0.79)	1.83 (±0.92)	0.268

^1^ SD, standard deviation.

## Data Availability

The datasets used and analyzed during the current study may be obtained from the corresponding author upon reasonable request.
